# Dermatologic manifestations of acute graft versus host disease after liver transplantation: A case series of 8 patients

**DOI:** 10.1016/j.jdcr.2023.06.035

**Published:** 2023-07-06

**Authors:** Manjit Kaur, Navdeep Singh, Rohan Mital, Austin Schenk, Kristopher Fisher, Abraham M. Korman, Benjamin H. Kaffenberger, Catherine G. Chung

**Affiliations:** a Department of Dermatology, The Ohio State University Wexner Medical Center, Columbus, Ohio; b Division of Transplant Surgery, Department of Surgery, The Ohio State University Wexner Medical Center, Columbus, Ohio; c Department of Pathology, The Ohio State University Wexner Medical Center, Columbus, Ohio

**Keywords:** acute graft-versus-host disease, liver transplantation, organ transplantation, solid organ transplantation, skin manifestations

## Introduction

Acute graft versus host disease after liver transplantation (aGVHD-LT) occurs when donor lymphocytes within the transplanted liver expand, recognize, and destroy the recipient tissue. While the incidence of aGVHD-LT is 0.5% to 2%, mortality is nearly 100%,^1^ underscoring a need for early detection and intervention by dermatologists. aGVHD-LT symptoms are similar to aGVHD after hematopoietic stem cell transplantation (HSCT), with the exception that liver function tests are normal in aGVHD-LT and the typical development of pancytopenia as part of the disease in aGVHD-LT.^1^

Skin rash is the most common clinical feature of aGVHD-LT (92%) and is typically one of the earliest signs of disease.^2^^,^^3^ Dermatologic manifestations of aGVHD-LT resemble adverse drug reactions or viral exanthems,^4^ both of which are common in the post-transplant period and are difficult to distinguish from aGVHD-LT. Here, we describe 8 cases of aGVHD-LT in our institution with emphasis on risk factors and dermatologic description to aid dermatologists in rapid identification of this potentially fatal complication.

## Methodology

The internal database of the comprehensive transplant center at The Ohio State University Wexner Medical Center was searched for liver transplant recipients who developed aGVHD-LT. Out of 1095 adult liver transplants performed between January 1, 2001 and January 11, 2022, all cases that had documented skin manifestations of aGVHD were included in this study. Recipient and donor data were extracted via chart review and analyzed quantitatively.

## Results

Of 1095 adult liver transplants performed between January 1, 2001 and January 11, 2022, 10 recipients (0.91%) developed aGVHD-LT, and 8 had skin manifestations. Recipient and donor demographics, clinical presentation, and outcomes are summarized in Table I. The indications for liver transplant included cirrhosis due to alcoholic liver disease (*n* = 3, 37.5%), nonalcoholic steatohepatitis (*n* = 2, 25%), hepatitis C (HCV) with hepatocellular carcinoma (*n* = 1, 12.5%), cryptogenic cirrhosis with hepatocellular carcinoma (*n* = 1, 12.5%), and primary sclerosing cholangitis (*n* = 1, 12.5%). All patients received a deceased donor ABO compatible whole liver graft. One patient had a dual organ (liver and kidney) transplant. Donor–recipient sex mismatch was present in 50% of the cases (4/8). Median recipient age was 64 years (range, 50-70 years) and median donor age was 32.5 years (range, 16-57 years) in this series. The median age difference between recipients and donors was 36.5 years (range, 4-43 years). This age disparity was higher in patients who died (median 39.5 years; range, 16-43 years) versus patients who survived (median 6.5 years; range, 4-9 years). Five patients were HLA-mismatched at all 6 loci, and the remaining 3 patients were mismatched at 4-5 loci.

**Table I:** Recipient and donor characteristics, clinical presentation and outcome

Case No.	1	2	3	4	5	6	7	8
Recipient age (yr)	62	50	55	69	70	70	66	59
Sex	Male	Male	Male	Female	Female	Male	Male	Male
Race	White	White	White	White	African American	White	White	White
Sex match	Yes	No	Yes	No	Yes	No	No	Yes
Degree of HLA match	0/6	0/6	1/6	0/6	2/6	0/6	1/6	0/6
Donor age (yr)	19	46	16	35	54	30	57	19
Donor sex	Male	Female	Male	Male	Female	Female	Female	Male
Disparity of recipient/donor age (yr)	43	4	39	34	16	40	9	40
Primary disease	ALD, HCC	PSC Cirrhosis	NASH	ALD	HCV, HCC	NASH	Cryptogenic cirrhosis (sarcoid), HCC	ALD
Diabetes mellitus	No	No	No	No	No	Yes	Yes	Yes
Clinical presentation	Fever, skin rash, non-bloody diarrhea, leucopenia	Fever, skin rash, leucopenia	Fever, skin rash, pancytopenia with refractory thrombocytopenia	Fever, skin rash, leucopenia	Skin rash, leucopenia, diarrhea	Fever, diarrhea, skin rash, pancytopenia	Fever, skin rash, nausea, vomiting, diarrhea	Skin rash, diarrhea, cytopenias
Outcome	Deceased	Alive	Deceased	Deceased	Deceased	Deceased	Alive	Deceased
Cause of death	Sepsis	NA	Sepsis	Multi organ failure	Sepsis, gastrointestinal bleeding	Acute hypoxic respiratory failure	NA	Acute respiratory failure

*ALD*, Alcoholic liver disease; *HCC*, hepatocellular carcinoma; *HCV*, hepatitis C virus; *NA*, not applicable; *NASH*, non-alcoholic steatohepatitis; *PSC*, primary sclerosing cholangitis.

**Fig 1:**
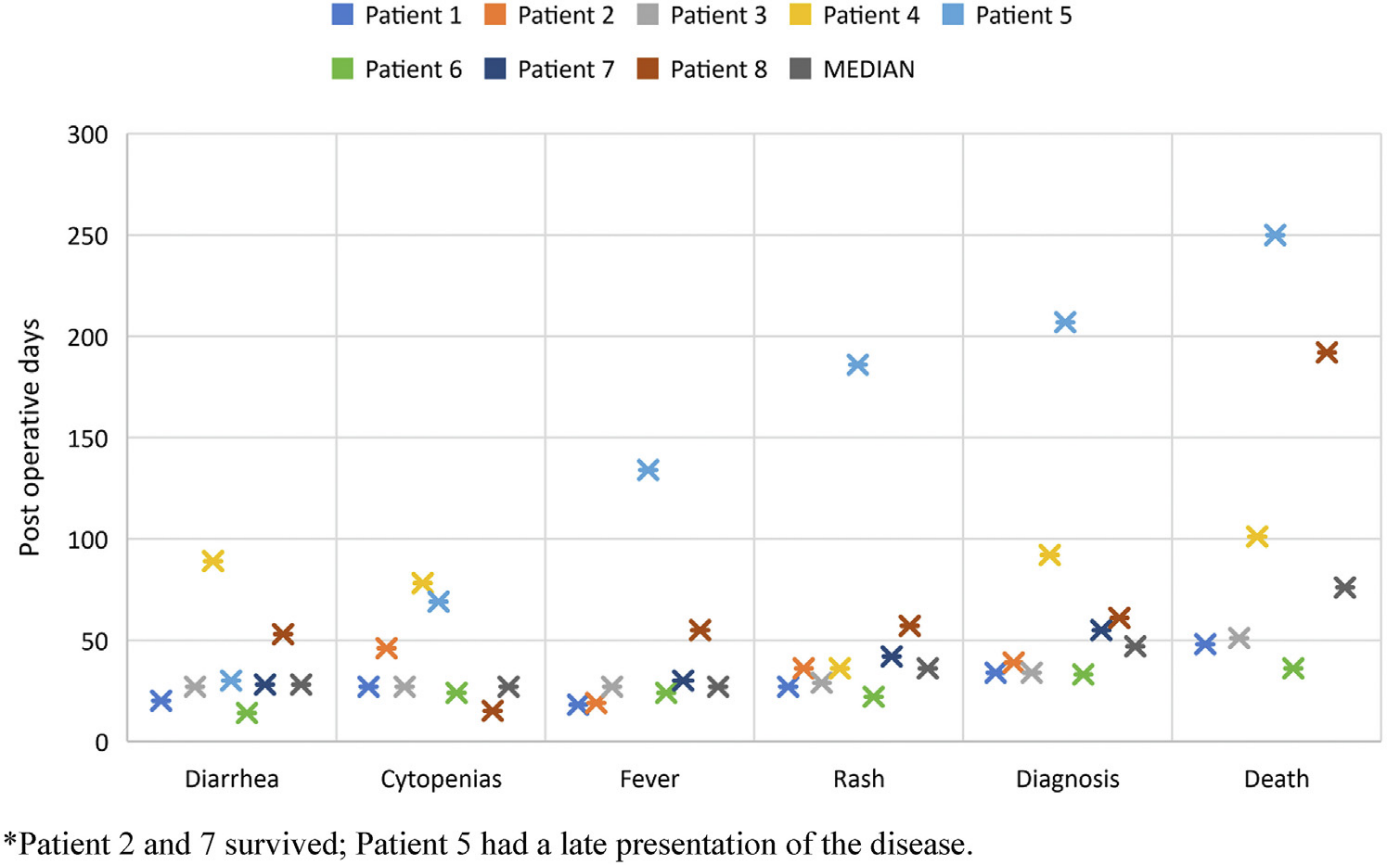
Acute graft versus host disease after liver transplantation - Graph showing timeline of clinical presentation, diagnosis and outcome.

Timelines for diagnosis, symptomatology, and clinical outcomes are shown in Fig 1. Time from transplantation to clinical presentation of aGVHD ranged from 14 to 134 days. Initial presenting symptoms were skin rash (*n* = 1), fever (*n* = 2), diarrhea (*n* = 4), and simultaneous fever and diarrhea (*n* = 1). Dermatologic manifestations are summarized in Table II. Skin rash appeared median 36 days post-transplant (range, 22-186 days). Most patients had a maculopapular rash, erythematous to dusky in color (*n* = 7), with perifollicular features in 2 patients. One patient developed Toxic epidermal necrolysis (TEN)-like involvement with desquamation and bullae formation (*n* = 1). The trunk was the most common area affected followed by proximal extremities > distal extremities, and legs > arms. Involvement of palms and soles was observed in 4 patients, face in 2 patients while oral mucosa and tongue involvement in only 1 patient.

**Table II:** Summary of dermatologic manifestations

	Case 1	Case 2	Case 3	Case 4	Case 5	Case 6	Case 7	Case 8
Average day of rash development (d)	27	36	29	36	186	22	42	57
Rash morphology	Maculopapular, non-blanching confluent rash, macules coalescing into patches, some fine scaling	Scaly scattered macules and papules	Generalized macular rash, desquamation, a few bullae	Diffuse erythematous to dusky macules and papules	Erythematous maculopapular rash, some morbilliform, a few hyperpigmented macules and papules	Erythematous macules and papules with superficial scaling, coalescing over flanks	Erythematous morbilliform, punctate rash	Perifollicular, morbilliform eruption, numerous purpuric lesions on legs
Sites involved	Chest, abdomen, back, a few lesions on legs	Face, ears, chest, abdomen, back, extremities (upper > lower)	Generalized, BSA>90%, worse on lower abdomen, lower back, face, palms and soles	Chest, abdomen, back and extremities	Chest, back, proximal arms, thighs, legs, palms and soles	Lower abdomen, suprapubic area, bilateral medial thighs, legs, lateral neck, upper chest, left flank	Chest, abdomen, back and proximal extremities with fewer lesions on distal extremities, palms and soles	Chest, abdomen, back, arms, legs
Mucosal involvement	No	No	Ulcers -oral mucosa, tongue	No	No	No	No	No
Pruritus	Yes	No	No	No	Yes	No	No	No

Diarrhea occurred in 7/8 patients post-transplant (median 28 days; range, 14-89 days). 7/8 patients presented with fever post-transplant (median 27 days; range, 18-134 days). Cytopenias were seen in 7/8 patients post-transplant (median 27 days; range, 15-78 days). The time from transplant to confirmation of the diagnosis of aGVHD-LT was median 47 days (range, 33-207 days).

All patients had pathological confirmation of aGVHD-LT. Results of the diagnostic work-up are summarized in Table III. Skin biopsy was performed in all cases at the time of rash presentation and demonstrated interface dermatitis consistent with aGVHD in all cases.

**Table III:** Results of the diagnostic work-up

	Case 1	Case 2	Case 3	Case 4	Case 5	Case 6	Case 7	Case 8
Chimerism in peripheral blood (CD3)	100% (Day 31)	0% (Day 37)	10% (Day 45)	93%(Day 87)	69% (Day 188) 87% (Day 199) 89% (Day 206) 81% (Day 223) 46% (Day 245)	78% (Day 25) 87% (Day 33)	13% (Day 51)	8% (Day 46), 14% (Day 60) 91% (Day 63) 86% (Day 69) 65% (Day 74) 64% (Day 77) 67% (Day 88) 60% (Day 95) 53% (Day 102) 53% (Day 109) 47% (Day 116) 100% (Day 158)
Chimerism in whole blood	93%	0%	21%	78%	0%	26%	0%	NA
Chimerism in bone marrow	94%	0%	27%	25%	0%	10%	0%	6%
Bone marrow (BM) biopsy	Hypocellular BM, trilineage hypoplasia	NA	Hypocellular BM (10%), granulocytic and erythropoietic hypoplasia	Variably cellular bone marrow (35%), granulocyte hypoplasia	Negative	Hypocellular BM (5%), trilineage hypoplasia	NA	Hypocellular BM (<10%), trilineage hypoplasia
FISH analysis		Positive on skin biopsy, scoring % cells with XX pattern: 15%		Positive on skin biopsy, scoring % cells with XY pattern: 37%		Positive on skin biopsy, scoring % cells with XX pattern: 30%		
Grade of GVHD	Grade 1-Skin, Grade 1-Intestinal	Grade 1-Skin	Grade 4-Skin, Grade 3-Intestinal	Grade 2-Skin	Grade 1-Skin	Grade 1-Skin	Grade 2-Skin	Grade1-Skin, Grade 0-Upper GI, Grade1-Lower GI

**Fig 2:**
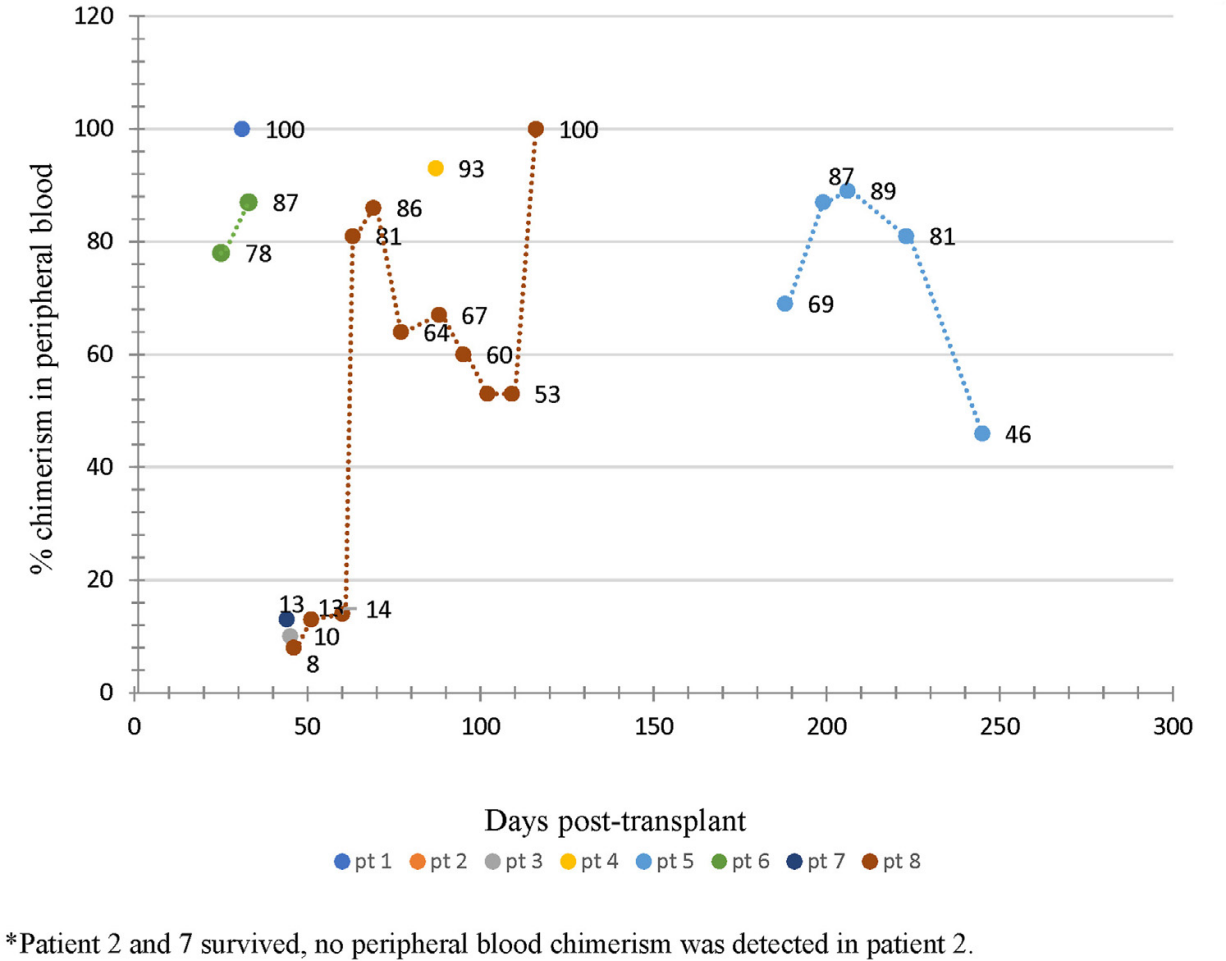
Acute graft versus host disease after liver transplantation - Graph showing chimerism values in the peripheral blood and their trend.

Chimerism by short tandem repeat polymerase chain reaction (PCR) assay was performed in all cases in the peripheral blood and bone marrow, and repeat levels were measured to monitor disease progression and response to treatment. Chimerism values in peripheral blood are shown in Fig 2. Blood-chimerism was identified in all patients except 1 (Case No.2), where aGVHD-LT was confirmed by FISH testing on the skin biopsy specimen. Histologically, cutaneous aGVHD-LT was documented as grade 1 in 5 patients, grade 2 in 2 patients, and grade 4 in 1 patient. Colon biopsy was performed in 3 patients and was graded histologically as grade 1 in 2 patients and grade 3 in 1 patient. Marked hyperferritinemia was seen in all the patients and the difference in peak ferritin levels in patients who died (mean 2662 ± 2173 SD) ng/mL vs who survived (mean 405 ± 536 SD) ng/mL aGVHD-LT was evident. This difference was not significant statistically (*P* = .215).

Most patients were treated with reduction in steroid-sparing immunosuppression, and in most cases calcineurin inhibitors and mycophenolate were stopped while patients were continued on corticosteroids. Increased corticosteroid doses were subsequently used in the treatment of aGVHD in all patients, etanercept in 3 patients, and anti-thymocyte globulin (ATG) and intravenous immunoglobulin (IVIG) were used in 1 patient each.

Six out of 8 patients died. Patients who survived aGVHD-LT were treated with only steroids. The mortality rate was 75% in this cohort, on median 76 days (range 36-250 days) after transplant. The cause of death was attributed to sepsis in 3 patients (50%), acute hypoxic respiratory failure in 2 patients (33.3%), and multiorgan failure in 1 patient (16.7%). None of the patients in this study received HSCT as treatment. Two patients were discharged after successful treatment and are alive to date.

## Discussion

aGVHD-LT is an infrequent complication, and the incidence of 0.91% found in our study is comparable to published reports.^1^^,^^2^ aGVHD-LT presents a diagnostic challenge. Final diagnosis was made by primary team and dermatology consultation was sought at the time of rash appearance in all patients.

In our study, the age difference between recipients and donors was markedly different in those that survived (median 6.5 years; range, 4-9 years) and those who died (median 39.5 years; range, 16-43 years). Donor recipient age difference of more than 20 years has been described as an independent risk factor for aGVHD-LT in the literature,^5^ and our results are supportive. Human leukocyte antigen (HLA) matching has also been described as a risk factor for aGVHD-LT, as HLA-matched donor lymphocytes easily escape the host immune system. Surprisingly, all patients in our cohort had a high degree of donor–recipient HLA mismatch. 3/8 patients (37.5%) in this study had alcoholic liver disease (ALD). ALD has been described as a risk factor for development of aGVHD-LT.^6^ Three (37.5%) of our recipients had a history of diabetes, which has also been described as a risk factor.^6^ However, 1 of these 3 patients developed a mild form of aGVHD-LT and survived.

Most patients in this study presented on median 36 days (range, 22-186 days) post-transplant with skin findings, which is longer than typically seen in hematopoietic stem cell transplant (HSCT) likely resulting from delayed engraftment of donor lymphocytes.^1^ One patient in this study had late onset disease at 134 days post-transplant. A similar late presentation of aGVHD-LT has been reported previously,^7^ however this is rare. Skin rash has been described as one of the earliest and most common clinical feature of aGVHD-LT,^2^^,^^4^^,^^8^ and in this study appeared on average 54.3 days, which is similar to 48.3-day average reported previously.^4^

The dermatologic manifestations in aGVHD-LT have been previously described as erythematous to violaceous, rapidly spreading symmetric morbilliform exanthems, or folliculocentric papules.^4^^,^^9^All patients in our study had a maculopapular rash and 2 of these patients had punctate and perifollicular lesions. In 1 patient, the rash started as a maculopapular eruption and later progressed to involve >90% BSA with desquamation, mucosal ulceration, and bullae, resembling toxic epidermal necrolysis. A similar presentation of aGVHD-LT has been described previously by Jeanmonod et al.^10^

The skin rash of aGVHD-LT may be pruritic^11^ or can be subtle and nonpruritic.^2^ Only 2 patients in our study had pruritus associated with their rash. Oral ulceration and mucositis were seen in only 1 patient who had TEN-like presentation contrasting a previous report with higher levels.^12^

Three patients (37.5%) in our cohort were initially misdiagnosed with infection or drug eruption. In 2 patients, skin biopsy was also equivocal initially to differentiate aGVHD-LT from other causes. In such cases, comprehensive drug history and viral serology testing may be useful. However, the presence of donor chimerism will confirm the diagnosis of aGVHD-LT.

Other common features of aGVHD-LT include fever, diarrhea, and cytopenia. Fever and cytopenias appeared on median 27 days and diarrhea presented on median 28 days after transplant in our cohort. These symptoms may be attributed to infections or immunosuppressive drugs, often leading to a delay in diagnosis.

In this study, skin pathology revealed interface dermatitis in all patients. Five patients had grade 1, 2 patients grade 2, and 1 patient had grade 4 cutaneous aGVHD-LT at the time of diagnosis.

Detection of chimerism is an important tool for the diagnosis of aGVHD-LT, in particular, measuring it sequentially to detect an increase. For diagnosis, it is important to establish the engraftment of donor lymphocytes in the recipient, and PCR is used to identify donor HLA in recipient bone marrow or peripheral blood. Donor engraftment is present if >1% of donor T-cells are detected, while persistent levels of >10% are indicative of aGVHD.^13^ The level of donor chimerism may correlate with the severity of aGVHD-LT manifestations. In our study, chimerism with the presence of >10% donor T-cells was detected in 7/8 patients. Of 2 surviving patients, 1 had no peripheral blood chimerism, and the other had 13% donor T-cells. The trend of chimerism in this cohort (Fig 2) further suggests that the increase and persistence of chimerism may be associated with increased severity of the disease.

FISH analysis may also detect the presence of donor cells and help confirm the diagnosis of aGVHD-LT, but its usefulness is limited by the fact that it can only be used when the recipient and donor are of the opposite sex. FISH has been used to confirm the diagnosis of aGVHD-LT in multiple patients whose peripheral blood short tandem repeat (STR) chimerism failed to show any donor lymphocytes.^14^

After a diagnosis of aGVHD-LT is confirmed, the goal of treatment is to suppress activated donor lymphocytes. Therapeutic approaches such as modified immunosuppression and agents such as anti-thymocyte globulin, IL-2 antagonists, TNF alpha inhibitors, or JAK1/2 inhibitors have been tried with varied success, but mortality remains high.^2^ Evidence-based guidelines for treatment of aGVHD-LT are lacking, and steroids remain the mainstay of treatment.^1^ Notably, steroid responsiveness is less in aGVHD after LT than after HSCT.^15^ In our cohort, the immunosuppressive drugs tacrolimus and mycophenolate mofetil (MMF) were withheld in all the patients followed by treatment with steroids. Despite aggressive treatments, 6 patients (75%) in our study died due to overwhelming sepsis, hypoxic respiratory failure, and multiorgan failure after the development of pancytopenia. This occurred median 76 days (range 36-250) after transplant, and median 15.5 days (range 3-131 days) after diagnosis. The time to diagnosis is considered an important prognostic factor in aGVHD-LT,^8^ and physicians should have a high index of suspicion to minimize delay. Mortality was very high in our cohort (75%), reinforcing existing reports. Two surviving patients had grade 1 and grade 2 aGVHD-LT, respectively, and they responded to oral steroids along with lowering of steroid-sparing immunosuppressive agents.

## Conclusion

Physicians should suspect aGVHD-LT in any liver transplant patient presenting with a maculopapular rash postoperatively. The rash of aGVHD-LT may mimic rash from a drug eruption or viral exanthem, and a skin biopsy is a simple procedure with low morbidity which may help establish the diagnosis. Key clinical and pathological findings are useful in identification of aGVHD-LT, and descriptions provided in this series may aid dermatologists in identifying this potentially fatal complication. The presence of fever, diarrhea, and pancytopenia along with skin rash may also suggest aGVHD-LT, however peripheral blood chimerism studies, FISH analysis, and tissue pathology are needed to confirm clinical suspicion. Further work must be done to identify patients at risk of aGVHD-LT and to establish rigorous diagnostic and treatment guidelines.

## Conflicts of interest

None disclosed.
